# First Survey on Sea Turtles’ Interactions in Mussel Farms in Italy

**DOI:** 10.3390/ani15192909

**Published:** 2025-10-06

**Authors:** Ludovica Di Renzo, Giulia Mariani, Marco Matiddi, Cecilia Silvestri, Stefania Chiesa, Tommaso Petochi, Giovanna Marino, Federica Pizzurro, Simone Fazio, Emanuela Rossi, Giuseppe Prioli, Ike Olivotto, Giorgia Gioacchini

**Affiliations:** 1 Istituto Zooprofilattico Sperimentale dell’Abruzzo e del Molise Giuseppe Caporale, 64100 Teramo, Italy; g.mariani@izs.it (G.M.); f.pizzurro@izs.it (F.P.); e.rossi@izs.it (E.R.); 2 Centro Studi Cetacei APS—E.T.S., 65125 Pescara, Italy; 3 Italian National Institute for Environmental Protection and Research (ISPRA), 00144 Rome, Italy; marco.matiddi@isprambiente.it (M.M.); cecilia.silvestri@isprambiente.it (C.S.); stefania.chiesa@isprambiente.it (S.C.); tommaso.petochi@isprambiente.it (T.P.); giovanna.marino@isprambiente.it (G.M.); 4 Istituto per lo Studio Degli Impatti Antropici e Sostenibilità Ambiente Marino (IAS-CNR), Sa Mardini, 09170 Oristano, Italy; simone.fazio@ias.cnr.it; 5 A.M.A. Associazione Mediterranea Acquacoltori, 00144 Rome, Italy; gprioli@coopmare.com; 6 Department of Environmental and Life Science (DISVA), Polytechnic University of Marche, 60131 Ancona, Italy; i.olivotto@staff.univpm.it (I.O.); giorgia.gioacchini@staff.univpm.it (G.G.)

**Keywords:** mussel farming, sea turtle, wildlife–aquaculture interactions

## Abstract

**Simple Summary:**

Loggerhead turtles (*Caretta caretta*), opportunistic marine feeders, are increasingly reported as a source of disturbance to mussel farming in the Adriatic Sea. This study combined a survey of Italian mussel farmers with gastrointestinal analyses of stranded turtles along the Adriatic and Tyrrhenian coasts, focusing on Mediterranean mussels (*Mytilus galloprovincialis*). Sea turtle sightings were most frequent in northern Adriatic regions (Veneto, Emilia-Romagna) during summer and in southern areas (Molise, Puglia) during autumn, likely linked to seasonal water temperatures. Mussels were the most commonly ingested mollusk in the Adriatic, with their presence in turtle diets increasing from 2018 to 2021. While not a primary prey, mussels appear to be a consistent dietary item among turtles due to adaptive feeding. The persistence of such interactions poses management challenges for mussel farms. Broader national and international assessments are recommended to evaluate and mitigate the impact of sea turtles on Mediterranean shellfish aquaculture.

**Abstract:**

Sea turtles, particularly the opportunistic feeder species loggerhead turtles (*Caretta caretta*), are increasingly reported as a source of disturbance to mussel farming operations, especially in the Adriatic Sea. Despite the evident damage caused by these interactions, comprehensive national data on the phenomenon are still lacking. This study aimed to address this gap through a survey conducted among Italian mussel farmers, combined with the analysis of gastrointestinal contents from stranded sea turtles along the Adriatic and Tyrrhenian coasts, focusing on the ingestion of Mediterranean mussels (*Mytilus galloprovincialis*). Survey results revealed frequent turtle sightings in the northern Adriatic (Veneto and Emilia-Romagna) during summer months (June to August), while southern regions (Molise and Puglia) reported more sightings in autumn (September to October), likely influenced by seasonal water temperatures. The Mediterranean mussel was identified as the most commonly ingested mollusk in the Adriatic, with a notable increase in presence from 2018 to 2021. Although mussels are not a targeted prey, they appear to be a consistent dietary component due to adaptive feeding behavior. These interactions are increasingly and consistently reported, leading to significant management challenges for mussel farms. Based on these findings, a broader national and international assessment is recommended to evaluate the overall impact of sea turtles on shellfish aquaculture in the Mediterranean.

## 1. Introduction

The global expansion of the fishery industry, coupled with the impacts of climate change, has significantly altered the availability of wild fish stocks, leading to changes in natural ecosystems and shifts in species distribution patterns [1,2,3]. Wild marine fish stocks have been steadily declining since 1980 [4]. Concurrently, the demand for aquatic and fishing products rose by approximately 3% annually between 1961 to 2019. Alongside a 1.6% yearly growth in the human population and the expansion of global trade, these factors have driven the rapid growth of the aquaculture sector [5].

Italy is the fourth largest aquaculture producer among European countries, with a national output of 129.746 tons in 2023 [6]. Bivalve shellfish represent a significant portion of the production, led by Mediterranean mussels (*Mytilus galloprovincialis*; Lamarck, 1819) (57.279 t) and Manila clams (*Ruditapes philippinarum*; Adams & Reeve, 1850) (21.547 t), as well as the still-limited production of Pacific oyster (*Magallana gigas*; Thunberg, 1793) (302 t), generating an estimated value of over EUR 285 million [7]. Concerning mussels, they are produced in most Italian coastal regions, mainly Friuli-Venezia Giulia, Veneto, Emilia-Romagna, and Marche regions on the Adriatic Sea. Other significant production sites are located in Puglia, on the Ionian Sea, and in the Liguria, Sardinia and Campania regions on the Tyrrhenian Sea [8].

The mussel farming systems adopted in Italy are the fixed system, the single-ventia long-line, and the multi-ventia longline (also known as Trieste long-line system). The former, with fixed poles, is adopted in lagoons or strictly coastal and sheltered areas. The single-ventia longline, a relatively recent system, is used in open sea areas; while the Trieste longline—developed in the 1980s—is used in sheltered areas. The mussel farms are therefore located in marine sites, ranging from a few meters deep to a maximum of about 40 m. Mussels are usually kept in nets at depths between 3 and 8 m [9]. The distance from the coast also varies considerably, from a minimum of 300 m to several nautical miles.

These factors should be considered when evaluating the interaction between aquaculture activities and marine wildlife, including sea turtles. Aquaculture sites provide a readily accessible food source for sea turtles, particularly loggerhead sea turtles (*Caretta caretta*; Linnaeus, 1758), which may consequently alter their feeding behavior and spatial distribution. Indeed, opportunistic feeding behavior by loggerhead sea turtles has been documented in the Mediterranean Sea [10,11].

Aquaculture sites provide a readily accessible food source for other marine species, which may consequently alter their feeding behavior and spatial distribution. It has been shown that true seals (*Phoca vitulina*; Linnaeus, 1758; *Halichoerus grypus*; Fabricius, 1791), bottlenose dolphins (*Tursiops truncates;* Montagu, 1821), cormorants (*Phalacrocorax carbo;* Linnaeus, 1758), gilthead sea bream (*Sparus aurata*; Linnaeus, 1758), grey herons (*Ardea cinerea*; Linnaeus, 1758), gulls (*Larus* spp.), pelicans (*Pelecanus* spp.), grebes (*Podiceps* spp.) and loggerhead sea turtles have been attracted by marine aquaculture facilities [12,13,14,15,16,17,18]. These species are attracted to aquaculture farms due to the availability of trophic resources [18,19,20], playing a role as biodiversity hotspots and artificial ecosystems [19,20,21]. However, the ecological effects of aquaculture are complex and involve interspecies interactions (e.g., predation and competition), human activities (e.g., breeding and harvesting), and environmental conditions [22]. The mixed trophic analysis (MTI) [21,23] suggests that aquaculture activities have a positive impact not only on zooplanktonic fish, cormorants, bottlenose dolphins, and common mullet but also on the reduction of greenhouse gas emissions [24]. Nevertheless, predator–farm interactions can lead to significant negative impacts, including economic losses for farmers and threats to wildlife health. Marine predators, such as seals, can cause production losses of 2–10% [25], while seabirds have been observed directly preying on fish within cages. Interactions with other species, such as dolphins, are harder to detect but remain relevant [26]. On the other hand, aquaculture operations can pose a risk for marine protected species in terms of habitat exclusion, entanglement, collisions, and behavioral changes [18].

In recent years, several shellfish farmers in Italy have begun to report episodes of mussel predation due to the presence of sea turtles, as already happened in Greek aquaculture farms. However, there are currently no scientific published data on this phenomenon. The only available data regarding sea turtles and aquaculture farms pertain to the risks of entanglement in aquaculture gear and the ingestion of discarded fishing and aquaculture materials [9,18]. The main species of sea turtles inhabiting the Italian sea water of the Mediterranean Sea are the loggerhead turtle *C. caretta* [27,28,29,30,31,32,33], the green turtle (*Chelonia mydas;* Linnaeus, 1758) [9,27,28,34,35,36], and the leatherback turtle (*Dermochelys coriacea*; Vandelli, 1761) [27,28,29,37,38]. Sea turtles exhibit diverse feeding behaviors depending on species, life stage, and habitat. Loggerhead sea turtles, for instance, are primarily carnivorous, feeding on benthic invertebrates such as mollusks, crustaceans, and echinoderms [39]. They forage mainly in neritic coastal zones where these prey often use their powerful jaws to crush hard-shelled organisms [40]. Recent studies have further investigated the feeding ecology of *C. caretta* in the Mediterranean Sea. Mariani et al. [10] analyzed the gastrointestinal contents of 150 sea turtles stranded or captured along the Adriatic and Tyrrhenian coasts of Italy. The results revealed dietary differences, including the consumption of Mollusca, between the Adriatic and Tyrrhenian Seas. Additionally, similar feeding behaviors were observed across life stages—juveniles, sub-adults, and adults—contradicting previous reports and indicating an opportunistic feeding strategy with high adaptability to available prey resources.

The present study aims to characterize the extent of interactions between sea turtles and mussel farms along the Italian coasts, as well as their potential effects on aquaculture activities, also in terms of possible economic loss. To achieve this, we combined online survey data from mussel farmers with feeding analysis from stranded sea turtle gastroenteric content. This study represents the first attempt to describe this phenomenon in Italy and provide useful data for implementing sustainable conservation and economic measures.

## 2. Materials and Methods

### 2.1. Survey on Sea Turtle Interactions Addressed to Mussel Farmers

An online survey was developed using Google Forms https://forms.gle/S9r8cJGnWB6EVpPW7 (accessed on 11 August 2025) and distributed to Italian mussel farmers through direct contact and with the support of the national shellfish farmers association (Associazione Mediterranea Acquacoltori—AMA) in 2023.

The data collection tool was structured into four sections:

1. General farm information, including: Email, Name of farm, Region; Scientific and common name of farmed species; surface of farming area (mq).
2. Presence of marine fauna: 13 questions for reporting the presence of other species within the farming area, including sea turtles, fishes, marine mammals, and birds (Table 1).
3. Sea turtle interaction: 10 specific questions aimed at collecting detailed information on interactions between sea turtles and mussel farm (Table 2).
4. Open-ended section: a space for additional comments, anecdotal reports, and optional upload of images that could help identify the sea turtle species and measures individuals (Tables S1–S4).

### 2.2. Sea Turtle Feeding Data Collection

Data on sea turtle feeding behavior from a previous study by Mariani et al. [10] were reworked for further investigations. The number of loggerhead sea turtle carcasses stranded or bycaught between 2018 and 2021 was used to calculate a frequency of occurrence (FO) per year and investigate the trend. For this specific study, particular attention was paid to the ingestion of *M. galloprovincialis*, and an FO was calculated over the years 2018–2021, based on the total number of sea turtle carcasses analyzed each year and the ingestion rate from Mariani et al. [10].

## 3. Results

### 3.1. Mussel Farmer Survey

**Figure 1:**
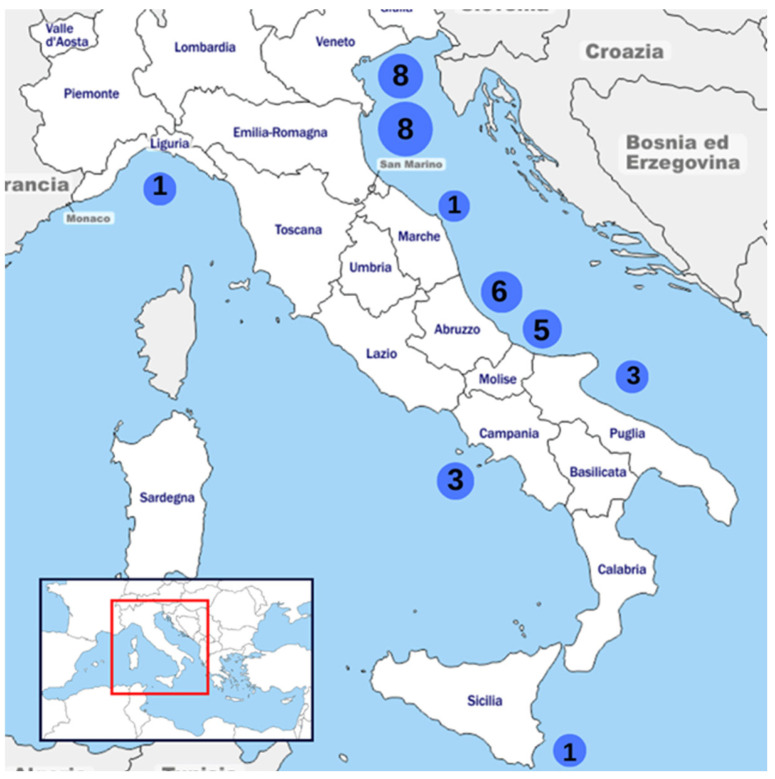
Geographical distribution of mussel farms that responded to the survey along the Italian coast. Each blue circle represents a region, and the number inside indicates how many farms per region responded, for a total of 36.

**Figure 2:**
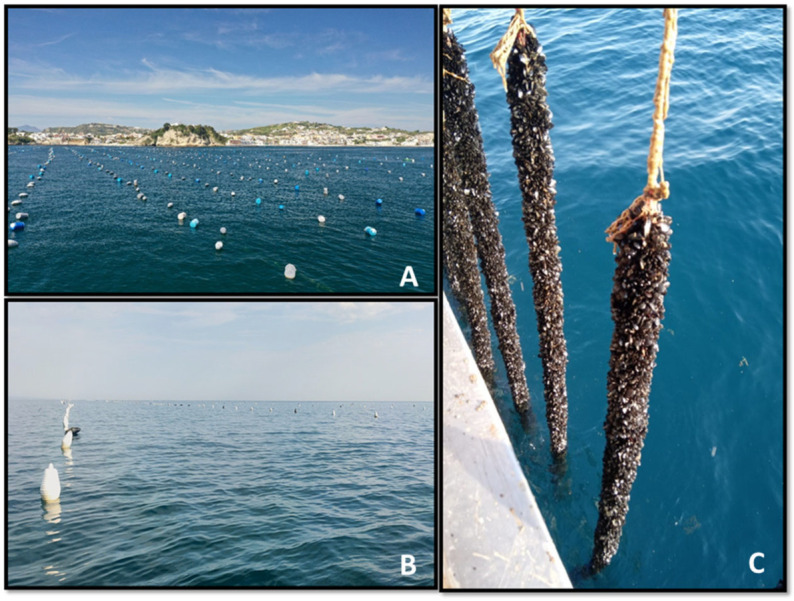
Single-ventia long-line in Italy view of the lands (**A**) and view of the open sea (**B**), long-line rafts hoisted out of the water (**C**). Photos by Matteo Ciani (ISPRA) & Ludovica Di Renzo (IZS).

Thirty-six Italian mussel farms from different regions successfully replied to the survey (Figure 1). All the farms use a multi-ventia long-line culture system (Figure 2) and the marine area managed by the mussel farmers ranges from 1000 m^2^ to 10,000,000 m^2^ (average 2,270,179 km^2^).

**Figure 3:**
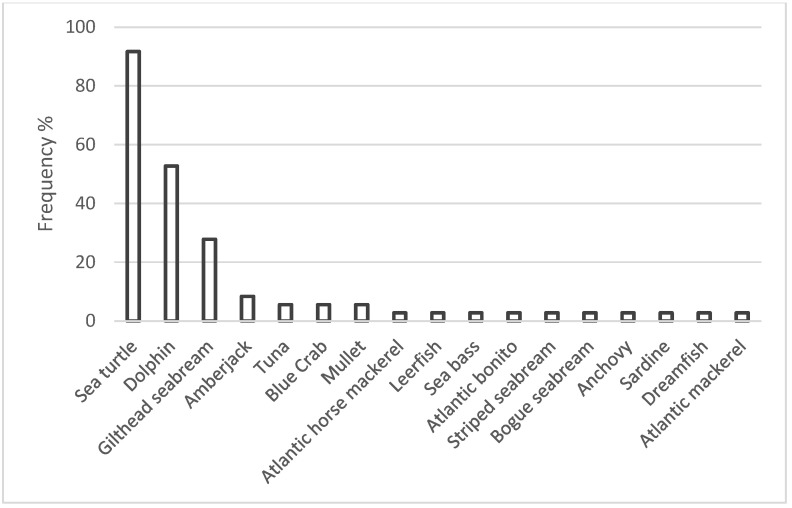
Frequency (%) of marine species spotted inside or nearby the mussel farming area by the interviewed farmers.

All of the mussel farmers declared that they had observed several marine species in the farm area, including sea turtles, pelagic fish, marine mammals and decapod crustaceans (Figure 3). Among the surveyed, sea turtles were reported as the main species which interact with the mussel farms (91.7%), followed by dolphins (52.8%), and gilthead sea bream (27.8%).

Among the 90% of farmers reporting the presence of sea turtles, 43.3% identified the species *C. caretta*, and, in only one case, *C. mydas*. Despite the technical support provided (Tables S1–S4), many mussel farmers (40%) stated that they could not distinguish between the turtle’s species. Eighty-one percent of the farmers reported having observed more than one individual at the same time, from a minimum of two up to ten as reported in one farming plant in Puglia (TA), reaching a maximum of 30 to 100 specimens at the same time reported in Emilia Romagna. Rarely (19%), only one specimen is spotted and reported.

The sea turtles observed ranged in size from 50 to 100 cm (average 78.1 cm), with the smallest individuals in size reported in Veneto (average 64 cm) and the largest in Emilia-Romagna (average 90 cm), followed by Molise (average 88 cm), Puglia and Campania (average 80 cm), Abruzzo (average 74 cm) and Marche (average 60 cm).

**Figure 4:**
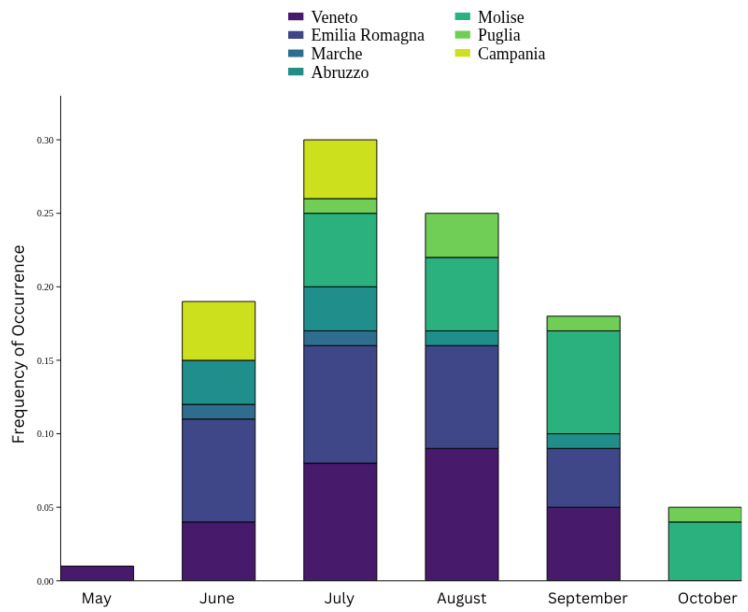
Frequency of occurrence of sea turtles spotted in mussel farms over the year. Seasonality of sea turtle interactions in mussel farms: southward tendency of sea turtles during the last part of the year.

Sea turtles are mostly observed during summer, with the highest frequency in June, July, and August. Specifically, in the northern Adriatic Sea (Veneto and Emilia Romagna regions), farmers usually spot sea turtles more often in July, August and September. In October, sea turtles are reported more southernly (Molise and Puglia) (Figure 4). In general, according to the survey, sea turtles are mostly observed in the morning (51.5% early morning, 45.5% late morning) and very rarely in the afternoon (3%).

**Figure 5:**
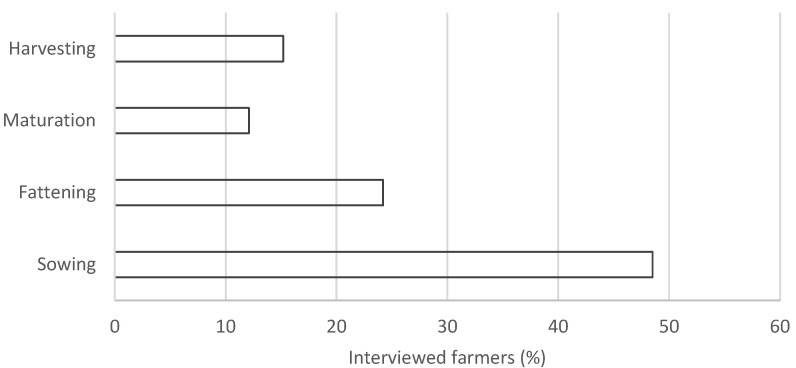
Phases of the production chain most affected by sea turtles’ interactions according to the interviewed farmers.

The survey found that few farmers (16.7%) have been observing sea turtles since the start of their farming activities, while most of them (66.7%) claimed that this phenomenon emerged in the last five years. Concerning the Adriatic regions, some companies have reported the phenomenon being present for over 10 years, while other companies in the same areas reported a time frame of 2–5 years. In general, 90% of the mussel farmers stated that, in recent years, there have been environmental changes that could have affected the farming activities. In particular, they all agreed that there has been an increase in water temperature. Although long-term Sea Surface Temperature (SST) data series are not available at mussel farms, an increase in SST data has been recorded by Copernicus CMEMS in the Adriatic Sea [41]. The data collected in the interaction section (Table 2) showed that the majority of farmers (91.7%), reported damage to the facility because of sea turtles. These included breaking of nets (socks, nets, rests, or braids) for 81.8%, whose contents are then dispersed (84.8%) or eaten (78.8%), as well as the deterioration of pergolas bitten by sea turtles and other species for the remaining 12.1%. Farmers reported the damage to the entire mussel farming area (72.7%), while, in 27.3% of cases, only one part is affected. The interactions occurred more frequently during the sowing phase (48.5%) compared to the last phases of fattening (24.2%), maturation (12.1%), and harvesting (15.2%) (Figure 5). The damage mentioned above has been estimated by 72.7% of mussel farmers to cause product loss of 1250 to 400,000 kg/year (average 87,729 kg/year), representing an economic loss of EUR 15,000 to 300,000 (average EUR 73,100). However, 33.3% of mussel farmers have never estimated damages in quantitative (kg) or in economic (USD/EUR) terms.

Only 13.8% of the mussel farmers have tried to adopt mitigation measures or dissuaders such as sound deterrents, protective stockings, and variations in the rearing bathymetry, but rare and small improvements have been observed for short periods. Such mitigation measures were not useful for these purposes: the protective stocking is only useful for the adult product, while the bathymetry variation was found to be unfavorable for growth. Moreover, the mussel farmers stated that the adoption of sound dissuaders was expensive for the company.

In addition to sea turtles, 27.8% of mussel farmers also reported the presence of gilthead sea bream, with sightings occurring in two cases: one in Liguria and one in Sicily, where it was the only species observed. The gilthead sea bream (4–6 kg in Liguria; 8–10 kg in Sicily) was primarily seen during summer evenings (starting in May) and was reported to be responsible for damaging the entire nursery along the water column, estimating a product loss of 461,664 kg per year. This phenomenon, which has been increasing, has been observed for approximately five years.

### 3.2. Sea Turtle Feeding Analysis

**Figure 6:**
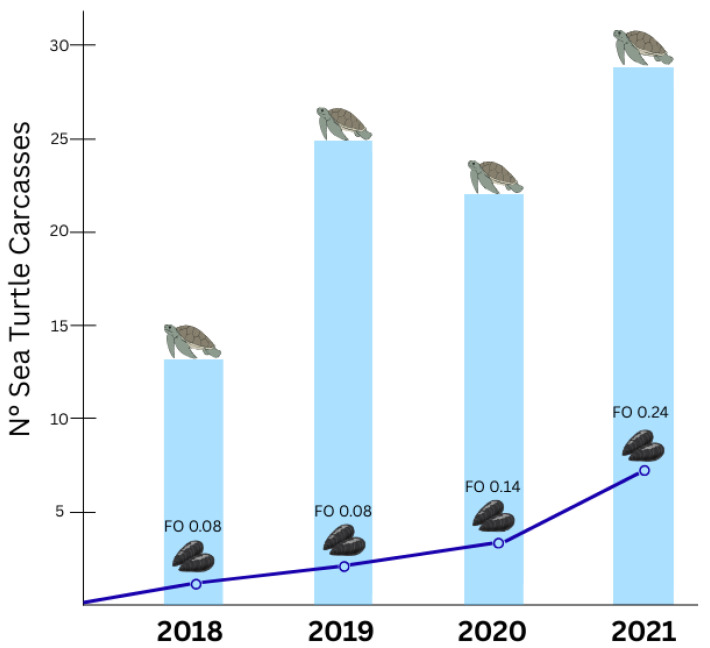
Ingestion of *Mytilus galloprovincialis* compared to the carcasses analyzed over the years (2018–2021). A frequency of occurrence (FO) refers to the *M. galloprovincialis* ingestion per year.

Among turtles that consumed mollusks, in the Tyrrhenian and Adriatic Seas, respectively, 62.5% and 54.1% fed on bivalves, with 14.61% and 6.56% specifically consuming *M. galloprovincialis* [10] (Table 3). The trend of the ingestion of *M. galloprovincialis* between 2018 and 2021 increased from FO 0.08 in 2018 to 0.24 in 2021 (Figure 6).

## 4. Discussion

Interaction between sea turtles and aquaculture has been documented in only a few studies worldwide, mostly focusing on entanglements in mussel farming equipment, such as longlines for mussel cultivation [18,31]. However, many cases are likely unreported [42]. This study constitutes the first investigation of interactions between sea turtles and mussel aquaculture along the Italian coasts, with a particular emphasis on the Adriatic region, motivated by heightened engagement from aquaculture farmers in this area.

Data collected from 36 mussel farms across Italy enabled us to evaluate the presence of sea turtles and other marine species with potential damage and disturbances to aquaculture infrastructures, providing quantitative and economic estimates of their impacts. Our findings identified *Caretta caretta* as the most frequently observed sea turtle species near the farms, consistent with its widespread presence in the Mediterranean Sea. In particular, the seasonal distribution of sea turtles in the northern Adriatic (Veneto and Emilia Romagna regions) mainly occurred during the summer months (June, July, and August); while the presence in the south (Molise and Puglia regions) was more frequent in September and October, suggesting a behavioral preference for warmer temperatures during the autumn season. This pattern supports previous studies reporting seasonal migrations of sea turtles to southern latitudes during winter [43], and their tendency to inhabit the northern and central Adriatic coasts in summer [44,45,46,47]. Specifically, adult males were observed migrating to Adriatic feeding grounds in May and June, followed by adult females in July and August.

Our dietary analysis offered further insights into the feeding ecology of *Caretta caretta*. Benthic mollusks are among the most commonly consumed prey items [32,34], with *Mytilus galloprovincialis* emerging as the most frequently ingested mollusk species by sea turtles in the Adriatic Sea. Notably, its presence in gastrointestinal contents increased steadily from 2018 to 2021: in 2018, the ingestion frequency of blue mussels in carcasses was 0.08, while, in 2021, it increased up to 0.24. Despite the number of carcasses increasing over the years, the number of blue mussels ingested also rose accordingly, suggesting a shift in feeding preferences due to availability. This preference, observed in an earlier study [48], was further supported by the discovery of stranded individuals whose stomach contents consisted exclusively of mussels [10]. Nevertheless, a broader dietary analysis [10] revealed that sea turtles primarily feed on arthropods (Table 3), suggesting that, while mussels are consumed, they are not a primary target food. Mussel farms, however, can act as biodiversity hotspots [49], attracting turtles that may feed on a variety of species, including both farmed mussels and associated fauna. This phenomenon appears to have intensified in recent years, and similar feeding behaviors have been reported in the Tyrrhenian Sea as well [10]. The presence of both adult and juvenile turtles in mussel farming areas suggests that these structures may serve as important attractants for individuals across multiple life stages.

Another noteworthy finding from our study is the detection of the invasive blue crab (*Callinectes sapidus;* Rathbun, 1896) in the stomach contents of sea turtles, with a frequency of occurrence (FO) of 1.12%. *C. sapidus* is now considered “virtually ubiquitous” in the Mediterranean Sea, according to Mancinelli et al. [50]. Until spring and summer 2023, the blue crab in Italy was mostly distributed in the Adriatic and Ionian seas, but, since then, the number of records and the reported abundance of blue crab along Italian coasts have been increasing [51,52]. This invasion profoundly altered local marine ecosystems, especially in the transitional environments along the Adriatic Sea, deeply affecting bivalve shellfish aquaculture [52]. It is well known that the blue crab represents prey of *C. caretta* in its natural area of distribution [53], but this represents valuable data for the Mediterranean. The low FO may be due to the time of sample collection and analysis, when the crab expansion was ongoing but not yet at the scale dimension that has been observed in the last two years. Therefore, the ingestion of blue crab by turtles not only highlights the species’ dietary plasticity but also serves as an ecological indicator of ongoing environmental transformations in the Mediterranean Sea, such as those driven by climate change and ecosystem disturbances.

This finding indicates that sea turtles are adapting to emerging food sources, including invasive species, reinforcing the notion that mussel farms and their surrounding areas are developing into biodiversity hotspots that attract a wide range of marine organisms. Notably, the analysis of dietary residues in sea turtles represents a valuable tool for ecological monitoring, offering key insights into ecosystem shifts and the proliferation of invasive species—an especially relevant approach in dynamic and rapidly changing environments such as the Adriatic Sea. From an economic perspective, the interactions between *C. caretta* and mussel farms result in significant damage, primarily due to the destruction of farming nets. Estimated losses range from 1250 to 400,000 kg of product per year, translating into economic damages between EUR 15,000 and EUR 300,000 annually. Despite the severity of these impacts, only a small proportion of farmers have implemented deterrent measures such as acoustic devices or seabed modifications. These interventions have shown limited and short-term effectiveness, emphasizing the need for more sustainable mitigation strategies.

Overall, rising water temperatures [54], which accelerate the sea turtle metabolisms [55,56], combined with overfishing and the spread of invasive species, have likely contributed to changing the habits and distributions of marine fauna, including sea turtles, as also reported by farmers in our survey. According to Copernicus, in Europe, the SST for 2019–2023 is 0.4 °C above the SST for 1991–2020. Moreover, temperature increases in marine coastal areas hosting aquaculture facilities in Italy have been observed over the last thirty years [23]. Based on model data from the Copernicus CMEMS service, the frequency of episodes with sea water temperature (−1 m deep) above 26 °C increased in the last decade in all Italian seas, particularly in Northern Adriatic Sea [41]. Such an increase in water temperature seems to be coherent with the main hypothesis given by most farmers who observed sea turtles in their facilities. This is followed by another hypothesis: a decrease in wild available food in the area for sea turtles. These factors, together with the transformation of mussel farms into ecological hotspots, highlight the complex interactions between aquaculture and marine biodiversity and underscore the importance of ongoing research in this field. For instance, it may not be a coincidence that sea turtles are increasingly being spotted also in transitional waters in Italy such as the Po Delta brackish lagoons [57,58], which are particularly vulnerable to climate change, as well characterized by numerous shellfish farms and an increased presence of blue crabs.

Therefore, climate change, particularly the rise in sea surface temperatures and the alteration of current patterns, may reshape the distribution and behavior of sea turtles [59,60]. Warmer waters may contribute to extending the seasonal presence of turtles in northern areas and influence their feeding ecology by facilitating the expansion of thermophilic and invasive species such as *C. sapidus* [61]. This environmental shift compels turtles to adapt to new dietary resources and alters their traditional migratory patterns, increasing their interaction with human activities such as aquaculture [52]. Understanding the cascading effects of climate change on these dynamics is crucial for developing effective conservation and management strategies aimed at mitigating human–wildlife conflicts in coastal ecosystems [62].

This study provides the first assessment of the interactions between sea turtles and mussel aquaculture along the Italian coast, particularly in the Adriatic Sea. Our results reveal that mussel farms, which increasingly serve as biodiversity hotspots, attract sea turtles of various sizes and stages of development, influenced both by the availability of natural prey and by ecosystem alterations such as the spread of invasive species and climate change. The feeding plasticity exhibited by *C. caretta* suggests a strong adaptive response to changing environmental conditions caused by rising sea temperatures and depletion of natural food sources. However, these interactions also pose substantial economic challenges for aquaculture operations, with significant product losses and the limited effectiveness of currently available mitigation measures.

## 5. Conclusions

This study marks a significant step toward understanding the interactions between aquaculture sites and marine fauna, with a particular focus on sea turtles. Given the economic and social relevance of the aquaculture sector in Italy, investigating these dynamics is essential for identifying preventive measures that safeguard both marine wildlife and aquaculture operations. The findings highlight the need for continuous ecological monitoring, such as the systematic recording of sea turtle interactions with mussel farms, including frequency, seasonality, and behavioral observations, as presented in the present study. This could be further enhanced by the implementation of image-based data collection systems (e.g., underwater cameras), which can provide additional details about turtle behavior and interactions. Developing such monitoring schemes is essential to informing effective strategies to mitigate sea-turtle-related damage in mussel farming areas, thereby indirectly supporting marine conservation. Additionally, the analysis of sea turtle diets has proven to be a valuable tool for monitoring marine biodiversity and assessing the ecological impact of invasive species. While aquaculture sites can be sources of conflict, they also have the potential to function as biodiversity hotspots. Further research is needed to balance ecological protection with economic sustainability, ultimately supporting conservation initiatives for endangered species such as *C. caretta* and enhancing the resilience of aquaculture in a changing marine environment.

## Figures and Tables

**Table 1:** Data concerning the species observed in the farm area and their characteristics. * Refers to the Supplementary Material for species identification (Tables S1–S3). ** Refers to the Supplementary Material for size determination (Table S4).

Species Observed in the Farm Area	
Do you observe other marine species in the farm area?	Yes/No
If yes, which species?	Open Answer
If you observe marine sea turtles, is the species *Caretta caretta*? *	Yes/Yes, but also *C. mydas*/Yes, but also *D. coriacea*/ No, it’s *C. mydas*/No, it’s *D. coriacea*/ No species/ I can’t recognize the species
Only one individual at a time?	Yes/Sometimes/No, always more than one/None
If you observe more than one individual in the same area at the same time, how many individuals you see (max)?	Open Answer
How big in size (cm) are the observed individuals? (In case of more than one individual, indicate more than one size) **	Open Answer
In which season this phenomenon is more evident?	Spring/Summer/Autumn/Winter
In which months this phenomenon is more evident?	Open Answer
In what time of the day this phenomenon is more evident?	Early morning/Late morning/Afternoon/Evening/Never
Have you always spotted them?	Yes, always/No, only from the last year/No, for few years/Never observed
If you have observed them for few years, how many years?	Open Answer
What could be the reason (or changes) behind the increasing presence of sea turtles in the farm?	There were no changes in the farm/Changes are not related to the farm but to the climate/There were changes, but never spotted sea turtles
Indicate the potential change responsible	Open Answer

**Table 2:** Data on the type of interaction.

Interaction	
Do sea turtles damage the farm?	Yes/No/There are no turtles
Which kind of damages they cause?	Open Answer
Are the damages limited to one area or is the farm entirely affected?	One part/the entire farm/No damages
If the damage involves only one area, what are the characteristics of this part?	Superficial areas/deep areas/water column/exposed area to the North/exposed area to the South/exposed area to the East/exposed area to the West/No damage
In which production phases sea turtles are observed to cause damage?	Open Answer
What is the extent of the damages (kg)?	Open Answer
What is the extent of the damages (€)?	Open Answer
Did you adopt any dissuaders/mitigation measures?	Yes/No
If yes, how many had a positive effect?	Yes/No/ Yes for a limited time/ Yes, partially/I didn’t adopt any dissuaders
What can be a solution?	Adopt expensive tools for the farm/I don’t know/I don’t need any solution

**Table 3:** Ingestion data from the study area (Adriatic and central Tyrrhenian Sea) with focus on the two main categories: Mollusca and Arthropoda (crustacean only). Data are given for two species of interest.

	Adriatic % FO	Tyrrhenian % FO
Arthropoda	94.38	37.70
*Callinectes sapidus*	1.12	0
Mollusca	62.92	83.61
Bivalvia	62.50	54.10
*M. galloprovincialis*	14.61	6.56

## Data Availability

The original contributions presented in this study are included in the article/Supplementary Material. Further inquiries can be directed to the corresponding author.

## Supplementary Materials

The following supporting information can be downloaded at: https://www.mdpi.com/article/10.3390/ani15192909/s1, Table S1: supporting images and information for the identification of *Caretta caretta*. Table S2: supporting images and information for the identification of *Chelonia mydas*. Table S3: supporting images and information for the identification of *Dermochelis coriacea*. Table S4: supporting images for the measurements.
