# Supplementary material for: First Survey on Sea Turtles’ Interactions in Mussel Farms in Italy
Source: Animals (Basel). 2025 Oct 6;15(19):2909. doi: 10.3390/ani15192909 (PMC12523913; doi:10.3390/ani15192909)
Supplement: Supplementary file 1 [file animals-15-02909-s001.zip › animals-3842835-supplementary.pdf]

Supplementary material

Table S1: supporting images and information for the identification of *Caretta caretta*.

Caretta caretta

Tartaruga comune

Loggerhead turtle

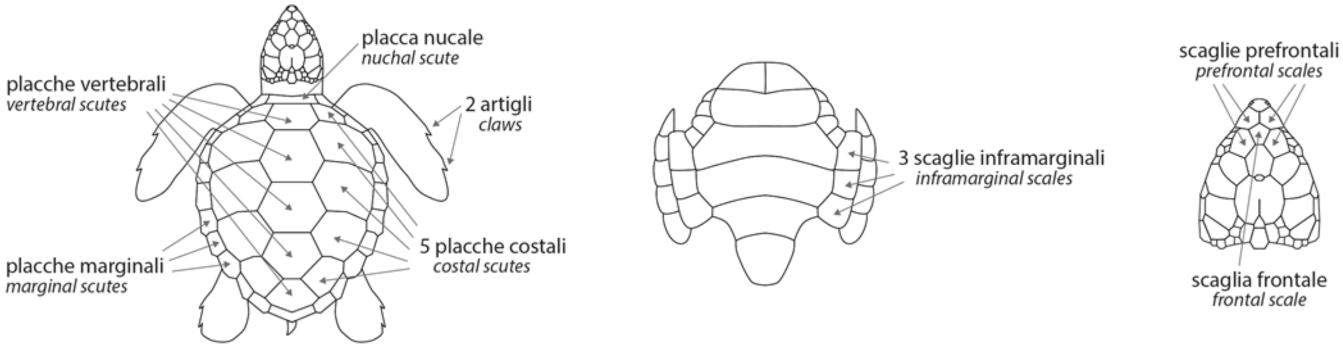

Colore/*Colour*	Rossiccio marrone/*Reddish brown*
Carapace/*Carapace*	Lunghezza massima 90-130 cm con una larghezza di 35-60 cm. Presenta 5 o più placche costali, la prima piccola tocca la placca nucale (o precentrale). *Maximum length 90-130 cm with a width of 35-60 cm. 5 or more costal plates, the first small one touching the nuchal (or precentral) plate.*
Piastrone/*Plastron*	Giallo arancione con 3 placche inframarginali. *Yellow-orange piastron with 3 inframarginal plates.*
Peso/*Weight*	100-160 kg
Testa/*Head*	Larga con due paia di scaglie prefrontali per lato. *Broad with two pairs of prefrontal scales on each side.*
Artigli/*Claws*	Presenta 2 artigli per arto e pinna. *Two claws per limb and fin.*

Table S2: supporting images and information for the identification of *Chelonia mydas*.

Chelonia Mydas

Tartaruga verde

Green turtle

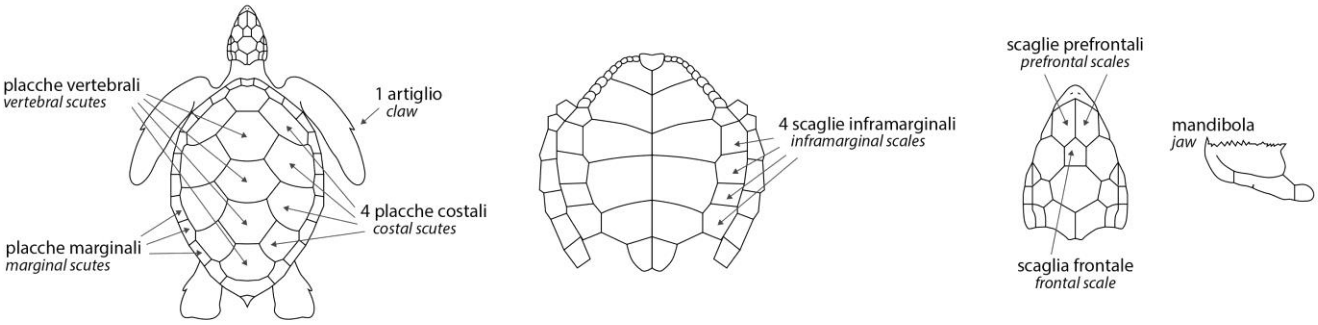

Colore/*Colour*	Nero nei neonati, verde oliva negli adulti. *Black in hatchling, olive green in adults.*
Carapace/*Carapace*	Lunghezza massima 90-125 cm con larghezza di 35-45 cm. Presenta 4 placche costali, la prima costale non tocca la placca nucale. *Maximum length 90-125 cm with a width of 35-45 cm. Four costal plates, the first costal does not touch the nuchal plate.*
Piastrone/*Plastron*	Giallo chiaro con 4 placche inframarginali. *Light yellow with 4 inframarginal plates.*
Peso/*Weight*	100-160 kg
Testa/*Head*	Tonda con la mascella seghettata. Un paio di scaglie prefrontali. *Round with a serrated jaw. A pair of prefrontal scales.*
Artigli/*Claws*	Un artiglio su ogni arto e pinna. *One claw on each limb and fin.*

Table S3: supporting images and information for the identification of *Dermochelis coriacea*.

<table>
<tr><td colspan="2" align="center">Dermochelys coriacea
Tartaruga liuto
Leatherback turtle</td></tr>
<tr><td colspan="2">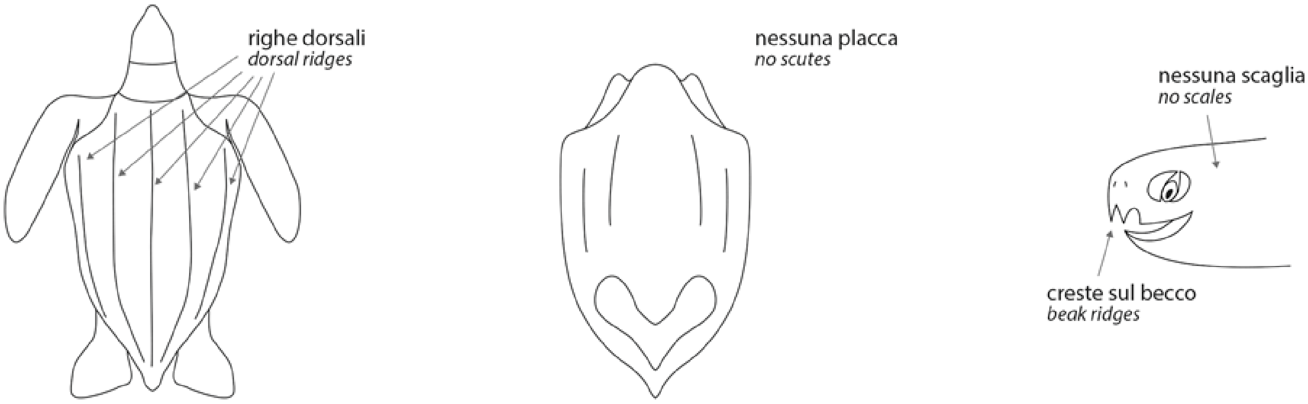
</td></tr>
<tr><td>Colore/Colour</td><td>Grigio scuro con qualche chiazza chiara. Dark grey with some light patches.</td></tr>
<tr><td>Carapace/Carapace</td><td>Lunghezza massima 165-195 cm con larghezza di 65-75 cm. Il carapace è simil cuoio con 5 linee longitudinali. Maximum length 165-195 cm with a width of 65-75 cm. The carapace is leather-like with 5 longitudinal lines.</td></tr>
<tr><td>Piastrone/Plastron</td><td>Relativamente piccolo. Relatively small.</td></tr>
<tr><td>Peso/Weight</td><td>400-800 kg</td></tr>
<tr><td>Testa/Head</td><td>Non presenta scaglie, con denti come creste su entrambi i lati della mascella. No scales, with teeth like ridges on both sides of the jaw.</td></tr>
</table>

Table S4: supporting images for the measurements

*CCL= Lunghezza curva del carapace (Dorso senza testa e collo) *CCL= Curved carapace length (back without head and neck)*
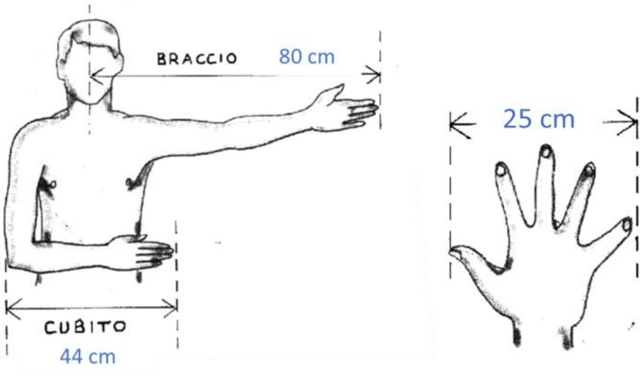
NB: Nel caso ci fossero più animali a dimensioni diverse segnare più di una misura.<br>*NB: If there are several animals of different sizes, mark more than one size*
